# Hospitals and Their Special Needs

**Published:** 1919-06-21

**Authors:** 


### HOSPITALS AND THEIR SPECIAL NEEDS.

**Belgrave Hospital for Children, Clapham Road S.W. 9.**—The hospital is situated in the centre of a district teeming with children, and, like other hospitals, is suffering from the increased cost of maintenance, and commenced 1919 with a deficit of £926. The total expenditure in 1918 was £6,200, as compared with £4,100 in 1913. The Committee are anxious to clear off this debt before the end of the year, and earnestly appeal for increased support to enable them to continue without interruption their work amongst the sick children of South-West London. In October last the babies' ward had to be closed for lack of nurses, and young ladies desirous of becoming nurses are invited to apply to the Matron, who has now a number of vacancies. Mr. Thos. Clapham is the Secretary.

**British Hospital for Mothers and Babies, and National Training School for District Midwives, Wood Street, Woolwich, S.E. 18.**—This institution hopes to begin to build, in the near future, on its three-acre freehold site in Woolwich, in view of the great national need of a Central College especially adapted to the requirements of pupils destined to work "on district." Donations to the Building Fund and also the Chapel Building Fund are urgently needed. Mr. A. C. Wickins is the Secretary.

**Central London Ophthalmic Hospital, Judd Street, W.C.** — This institution, founded 75 years ago and recently rebuilt, is equipped with every modern appliance for dealing with cases requiring treatment for the eye. It is greatly in need of increased support to enable it to deal with the large and growing numbers of patients, and, like many other hospitals, is feeling the difficulty of meeting the high prices of every commodity required. Having no endowments, annual subscriptions form the most reliable source of income, and the Committee are anxious to have the number of these increased, but they will be glad to receive support in any form to assist them in carrying on their work. Mr. H. R. S. Druce is the Secretary.

**Charing Cross Hospital, W.C.**—This hospital is now free from debt, but as it has very little endowment it is almost entirely dependent upon the generosity of the public for its continued maintenance. During the war the five wards which were previously closed for want of funds were used for the treatment of wounded sailors and soldiers, and in this way some 3,700 men have been dealt with and, in the majority of cases, restored to health and strength. A considerable part of the cost of these wards was, of course, defrayed by Government grant. Now these wards are to be kept open for the reception of civilian patients, and the whole burden of their cost must fall upon the hospital's funds.

**Chelsea Hospital for Women, Chelsea, S.W. 3.**—The Chelsea Hospital for Women is at the present moment, chiefly through the prevailing high prices, £3,000 in debt to its bankers. The work of the hospital is seriously entailed by the necessity of housing in half the wards the nurses and servants until the Nurses' Home can be built. For this purpose, a further sum of about £12,000 is needed. The work of the hospital concerns not only the health of women, but also indirectly the welfare of their homes and little ones. The Secretary, Mr. H. H. Jennings, will be grateful for contributions.

**Cheyne Hospital for Sick and Incurable Children, Chelsea,** is greatly in need of help, as a considerable deficit is shown in the accounts for the past year. The hospital has been kept quite full, and its branch hospital at St. Nicholas-at-Wade, Kent, was one of the very few institutions on the South-East Coast which remained open throughout the war. At the two institutions eighty patients are regularly treated, and several children, in addition, have been kept under supervision at country cottages to gain strength before being finally discharged. The Secretary will gratefully acknowledge any contributions that may be sent.

**Children's Hospital for the Treatment of Hip Disease, Sevenoaks.**—It is only at such special hospitals as this that sufferers from hip disease are admitted at the very beginning of the malady and kept until they are cured or become incurable, or, as in some cases, until released by death from their sufferings. Seventy-three children have been under treatment during 1918. Of these, twenty-five have returned to their homes cured or much improved. The financial position causes much anxiety.

**City of London Hospital for Diseases of the Chest, Victoria Park, E.** — The heavy increase—from £14,000 to £24,000 per annum—of ordinary maintenance expenditure at this hospital compels the Committee to plead with all to whom the voluntary system of hospitals is precious to assist them in some measure. Not until the general requirements are assured can the Committee concentrate upon the completion of most urgent structural improvements and additions which, unfortunately, had to be curtailed on the outbreak of the war. These improvements involve an expenditure of at least £15,000. Additional facilities for research work are also in contemplation. Contributions will be gratefully received by the Treasurer, or by the Secretary, at the hospital.

**City of London Maternity Hospital, City Road, E.C.**—Admissions to hospital, 6,196; number of births in hospital, 5,915; patients attended in their own homes, 2,703; number of births, 2,750; pupil midwives and nurses trained, 456; medical students taking midwifery course, 152; anti- and post-natal clinics held weekly. Owing to bank, £7,200. The Secretary is Mr. Ralph B. Cannings.

**East London Hospital for Children.**—This hospital is making urgent appeal for help. In commemoration of its jubilee a fund is being raised to provide a nurses' home and many other much-needed improvements. The work which this hospital is doing in the poorest part of East London calls for sympathy and support, for the nation's need for healthy children was never greater than now.

**French Hospital, Shaftesbury Avenue.**—It is the intention of the Committee to reconstruct and enlarge the building, and plans have been drawn up to this effect. Taking into consideration the increased cost of labour and material, the probable cost is calculated at £25,000, and all friends and benefactors are asked to help find the funds necessary for this undertaking.

**Hospitals and their Special Needs**—(*continued*).

**Guy's Hospital, S.E. 1.**—At this famous institution 8,331 in-patients and 96,877 out-patients received treatment in 1918, while 1,935 maternity patients were attended in their own homes. The endowments provide less than half the ordinary expenditure of over £108,000. There are practically no reserves, but the provision of increased accommodation for nurses and for massage, children's wards, and other special departments, planned before the war, can no longer be delayed. Any financial assistance, either to general maintenance or to this special object, will be gratefully welcomed. The Treasurer is Viscount Goschen.

**Hampstead General Hospital.**—There are 126 beds at Haverstock Hill, Hampstead, including 105 free, fifteen contributory at £2 2s. a week, and six private rooms at £4 4s. a week. There is also a well-equipped out-patient department at Bayham Street, Camden Town. The institution serves the whole of the North-West London district. A substantial addition to the income is urgently needed to enable the Council to carry on the work, which is much hampered by a debt on maintenance account of nearly £10,000.

**Hospital for Epilepsy and Paralysis, Maida Vale.**—Owing to its benevolent activities on behalf of soldiers, pensioned soldiers, and officers of the Royal Air Force, the hospital finds itself, after forty years of freedom from debt, with a deficit of about £5,000. Generous and immediate help to liquidate this debt is earnestly asked. Mr. H. W. Burleigh is the Secretary.

**King's College Hospital.**—During the past four years King's College Hospital has provided not only general, but special medical and surgical, treatment for patients, as follows: 12,000 civilian in-patients, 29,000 military in-patients, 117,000 new out-patients, and 537,601 attendances. £100,000 is urgently needed to free the hospital from debt, and £50,000 a year additional income to enable the Committee of Management to place at disposal, for benefit of civilian patients who need special medical and surgical treatment, that portion of the hospital shortly to be evacuated by the military. Mr. Richard J. Coles is the Secretary.

**London Homœopathic Hospital.** — The Board of Management are making special efforts to complete their appeal for £16,710; up to the end of 1918 £10,420 had been reached, and during the present year £2,054 has been received, leaving £4,236 still to be raised. A lady has promised £100 if five other donors, of either sex, will give a similar amount. Contributions addressed to the Treasurer—the Earl of Donoughmore—at the hospital, Great Ormond Street, W.C. 1, will be gratefully acknowledged. The Secretary is Mr. E. A. Attwood.

**London Hospital, Whitechapel,** with its 1,000 beds, is not only the largest hospital in England, but has to grapple with the endless needs of the most populous and poorest parts of London and the adjoining counties eastward. Needs and the "London," indeed, have become a permanent combination—the inevitable result of a singularly "live" hospital with a wholly inadequate endowment. It costs £200,000 a year to-day to run the charity efficiently, and towards this there is less than £40,000 of assured income. The balance—£160,000—must be sought from a generous public, and on its finding everything depends. To make good war attrition, to carry on, and to go forward are the three great needs of the "London" to-day. The hospital is not in debt, but the daily bill of £561 must be met if the "London" is to do what it can do.

**London Lock Hospital and Rescue Home.**—At the Female Hospital, Harrow Road, Paddington, a new maternity ward and labour unit has been reconstructed, and since it was opened in July 1918 eighty babies have been born, many of them free of venereal disease owing to the facilities offered for treatment of the mother in the early stages. A new children's ward has been started with seventeen beds. Over 40,000 men, women, and children receive treatment and assistance year by year at the male and female Lock Hospital. After allowing for the grant-in-aid from the Local Government Board, £12,000 per annum is required to maintain the institution under the control of the board. Contributions will be thankfully received by the Right Hon. Lord Kinnaird, K.T., Chairman of the Board of Management.

**Middlesex Hospital, W. 1.**—The hospital has for more than 170 years rendered inestimable benefits, not only to the sick poor of the densely populated neighbourhood in which it is situated, but to countless others, who nowhere else could have obtained that skilful treatment which is the marked characteristic of a well-equipped hospital. Some 5,000 patients are received into the wards each year, and in 1918 over 51,000 new patients (in addition to those already on the list) were treated in the out-patient department. Electrical and massage departments are also provided. In the cancer wing there are ninety beds, always occupied. The Secretary-Superintendent is Mr. Walter Kewley.

**National Hospital for the Paralysed and Epileptic, Queen Square, Bloomsbury, W.C.**—This hospital of 200 beds, with its branch at East Finchley, and the hospitals managed for the Ministry of Pensions at Maidenhead and Clapham, now treats 322 in-patients, this number including 112 discharged sailors and soldiers suffering from paralysis, epilepsy, brain spinal tumours, neuralgia, neurasthenia, neuritis, sciatica, St. Vitus' dance, and the many diseases of the nervous system, which for upwards of sixty years have been treated by the hospital. The manifestation of these diseases are often shattered nerves, blindness, deafness, and mutism. The hospital is, in addition, a well-known training centre for medical men, and men and women who are studying massage, electrical treatment, and remedial exercises. A sum of £30,000 is required annually from voluntary sources for the whole of this necessary and extensive work. The Treasurer is the Earl of Harrowby, and the Secretary Mr. Godfrey H. Hamilton.

**Paddington Green Children's Hospital.**—The hospital has forty-six beds, and has a convalescent home at Slough with twenty-four beds. The income last year from all sources was £5,928, expenditure £8,313; the deficiency of £2,385 had to be made good out of the hospital's slender resources. 711 in-patients received treatment during the year, and 13,874 new out-patients were treated. The out-patients' attendances number 4,122. The hospital is still doing its utmost to "save the babies." Secretary, Mr. F. Stanley Cheer.

**(Continued on page 311.)**

**Hospitals and their Special Needs**—(*Continued from page* 310.)

**Poplar Hospital for Accidents.**—The Committee were considering a scheme for the re-building of the west end of the hospital in 1914. This included a new and more commodious waiting-room for patients attending the casualty department, an *x*-ray operating room, with dark room, etc., and a waiting-room, a small operating theatre for casualties, and several other minor improvements—all absolutely necessary to bring the hospital up to date. About £19,000 has been allocated during the last five years for carrying out this scheme, which the Committee hope to commence when men and materials are available. Considerably more money will, however, be required, and a further appeal will be launched this year, the motto of the hospital being "never to be in debt," and never to spend any money it has not got in hand. Mr. Percy Rogers is the Secretary.

**Prince of Wales's Hospital, Tottenham, N.**—This institution depends entirely upon voluntary contributions for its support. No letters are necessary to secure the benefits which it administers. Over 30,000 patients are treated each year, and there are 125 beds to maintain. Fourteen thousand pounds is required annually to carry on the work of this hospital, which is situated in one of the most poor and needy districts of London, and a debt of £6,000 has yet to be liquidated. Subscriptions and donations will be gratefully acknowledged by the Director, Mr. Frederick W. Drewett.

**Queen Charlotte's Hospital, Marylebone Road, N.W.**—This hospital, the largest of its kind in England, receives about 1,800 patients into its wards yearly, and attends about the same number in their own homes. During the war more than 5,000 soldiers' and sailors' wives have been treated. The Committee are anxious to proceed with an extension of the hospital (new out-patient department) and important improvements in the wards, which have been postponed during the war. The cost is now estimated at £40,000, but the hospital is hampered with a debt of £10,000 accumulated during the war, which must be paid off before fresh liabilities are incurred. The maternity and child-welfare work of the hospital was never more important than at the present time, but additional support is greatly needed, for the expenditure is now double what it was before the war. The treasurer is Mr. Anthony de Rothschild, to whom donations may be sent, or to the secretary (Mr. Arthur Watts).

**Queen's Hospital for Children, Bethnal Green, E.**—It is absolutely necessary that the hospital should be considerably enlarged, and King Edward's Hospital Fund has allocated £1,250 towards a new out-patient department, conditionally upon an architect's certificate being produced within two years. A sum of £50,000 is needed. An appeal, signed by the mayors of the surrounding boroughs, states that every day's delay means death or unhealthy life to children who could be saved by the timely provision of proper facilities for their treatment, and appeals for help.

**Royal Free Hospital, Gray's Inn Road, W.C. 1.**—Notwithstanding the heavy additional cost of maintenance, the Royal Free Hospital has recognised the necessity for extending its maternity work, it being most essential that no opportunity should be neglected whereby improvements are effected in a matter of such vital public concern. With this object the maternity hospital hitherto maintained by the Duchess of Marlborough in Endsleigh Street, W.C., has now been incorporated with the hospital, and is known as the Marlborough Maternity Section. A further extension of the hospital has also been decided upon, and plans are in course of preparation for building additional wards on an adjoining site recently presented to the hospital. It is proposed to issue an appeal during the autumn for not less than £500,000.

**Royal London Ophthalmic Hospital, City Road, E.C.1.** The hospital is well known to the suffering poor, and it is famous in the medical world. But few people realise that every day it relieves over 300 out-patients and over 100 in-patients. The waiting-list is large, but eighteen beds still remain unoccupied for want of funds; and the hospital appeals for generous help to carry on its work of saving, or restoring, the priceless gift of sight.

**Royal National Hospital for Consumption, Ventnor.** Since this hospital was opened, just fifty years ago, over 30,000 in-patients have received the benefit of treatment in the beautiful Undercliff of the Isle of Wight, and of this number over 14,000 have been drawn from the Metropolitan area. Owing to the greatly increased cost of all supplies the necessary expenditure for maintenance is now some £5,000 per annum in excess of pre-war times.

**Royal Sea-Bathing Hospital, Margate.**—A new wing, to be called "King George the Fifth Wing," to contain sixty-eight beds, is being erected primarily for the reception of men discharged from His Majesty's Forces suffering with surgical tuberculosis, towards the cost of which the Local Government Board has made a grant of £10,880. This grant will by no means cover the cost, and at least a sum of £13,000 is required from the public to meet the difference and to pay the cost of providing equipment and additional accommodation for the increase in the staff which will be necessary. The beds will be available for the general public when not required for ex sailors and soldiers. The offices are at 13 Charing Cross, S.W., and Mr. A. Nash is the Secretary.

**Royal Westminster Ophthalmic Hospital, Strand, W.C. 2.**—Continuous support is needed to meet the claims upon this institution. Maintenance expenses still rule high, whilst the increases which have been made in salaries and wages to nurses, etc., further enhance the expenditure. A great deal of "spring cleaning" is also necessary this year, as on account of the high prices and lack of labour ruling last year, very little of this work took place.

**St. Andrew's Hospital, Dollis Hill, N.W. 2.**—The War Office has just given up the beds reserved for wounded soldiers and sailors, in which 2,430 British, Colonial, and Belgian men have been nursed. Warm praise has been received from the military authorities for the care of these wounded men. The opportunity of carrying out certain much-needed work, such as the provision of an observation block and the improvement to the servants' quarters, will entail an expenditure of nearly £1,200. Peace offerings towards this sum and for the maintenance of the hospital can be sent to the administrator, Mr. W. E. C. de Wiart.

**St. George's Hospital, S.W. 1.**—This hospital, which was established 186 years ago, is still carrying on the work then commenced for the sick poor, as may be seen from the fact that in 1918 4,254 in-patients and 29,073 out-patients received treatment. Of the former, 834 were sent to the convalescent branch at Wimbledon free of any cost to themselves. During the period of the war 1,983 soldiers and sixty-nine sailors were treated in the hospital, and the majority made a good recovery. The ordinary expenditure exceeded the ordinary income in 1918 by £19,059, and,

**Hospitals and their Special Needs**—(*continued*).

so far as can be ascertained at present there seems every likelihood of a similar deficit this year, so that the kind assistance of all who may have the welfare of this hospital at heart is earnestly desired. The Secretary is Mr. J. M. Churchfield.

**St. Mark's Hospital, City Road, E.C.**—The hospital is free—no charge whatever is made for treatment. It is now badly in need of funds—£4,000 is urgently required to pay off a loan and outstanding tradesmen's accounts. A number of very difficult cancer cases amongst soldiers have been successfully treated. Mr. H. W. C. Coope is the Secretary.

**St. Mary's Hospital, Paddington, W.2.**—Throughout the war the inoculation department of the hospital has supplied vaccines for the protection of the soldier against typhoid and other war diseases to all the allied armies in the field (British, Belgian, French, Russian, and Serbian). Upwards of thirteen million doses have been manufactured and supplied. The work has been acknowledged by the Army Council as "of great practical value to the troops in the field, as well as to the State." It is still going on, and has been the means of saving the lives of many thousand valiant men. Meanwhile the urgent claims of war funds were diverting the stream of financial support, and the ranks of annual subscribers suffered losses amounting to £1,000 a year. The hospital now seeks to re-establish the depleted ranks of its subscribers, and to find £13,000 to meet the expenditure of 1919.

**St. Mary's Hospital for Women and Children, Plaistow, E. 13.**—At the end of May the hospital owed its bankers £1,090. This may be considered satisfactory when it is realised that through the years of the war no beds have been closed, and the work carried on, so far as the patients were concerned, to the fullest extent possible. During these years many valuable subscribers have been lost, and it is only due to a legacy that the financial position is as good as it is. Similar revenue cannot be hoped for during this year, and new subscribers and donors will be required to help to meet the increased cost of maintenance.

**St. Thomas's Hospital.**—This institution is suffering very severely as a result of the war. It has for many years enjoyed a substantial income of from £60,000 to £65,000 per annum, derived from its dividends and rents, and this represented about two-thirds of its total expenditure. The balance of income is made up by a somewhat limited number of subscribers, from donations, and from legacies. Directly war broke out 200 beds were placed at the service of the Army and Navy, and in March 1915, at the request of the Army authorities, the military section of the hospital was greatly extended, and the total number of beds in the hospital was raised to 1,014. The expenditure was enormously increased, and owing to the extra number of beds maintained and the increased cost of running the institution the total outlay in the year 1918 was £145,505. There was no increase in the returns from invested property, and the total income was £122,833. The amount received for the treatment of soldiers was less than one-third of the expenditure, although the soldiers represented half the total number of patients resident in the wards. The number of officers, N.C.O.s, and men admitted to and treated in the wards between August 1914 and the end of December 1918 was 10,921. St. Thomas's is still carrying on very important work in the treatment of war pensioners and of limbless officers, for whom eighty beds are reserved. The average number of beds occupied per diem in 1918 was 851. Altogether 9,234 patients were admitted during the year, and 55,073 out-patients were treated. The expenses of maintaining the hospital still remain abnormally high, and income must be provided unless the work is to be seriously curtailed. This would be a calamity for the poor of the South-East of London, who have learned to depend on St. Thomas's. We feel that the pioneer work in the care of mothers, of babies, of the whole social side of hospital work, in the treatment of tuberculosis, in the splendidly organised physical-exercise department, in the speech clinic, and other special branches of work is so well known that we have but to make it clear that funds are sorely needed to carry on the work to make everyone realise how important it is to help St. Thomas's, one of the most ancient hospitals and charitable endowments in London. Donations and subscriptions are urgently needed, and legacies are earnestly appealed for.

**Samaritan Free Hospital for Women, Marylebone Road, N.W. 1.**—To retain to the suffering women of the United Kingdom the benefits which this hospital creates and which go so far towards the alleviation of suffering and distress, the Committee of Management earnestly plead for £3,000 to meet overdue tradesmen's accounts and to pay off the bankers' loan of £1,000. During the past five years the work of the hospital has been carried on under great difficulties, yet the Committee are proud to say that not a single bed has been closed. On behalf of poor and suffering women the Committee earnestly appeal for help. The secretary is Mr. G. H. Hawkins.

**Seamen's Hospital, Greenwich.**—During the four years of war the wards of the Seamen's Hospitals have been full, the patients including both men of the Royal Navy and the equally gallant men of the Merchant Service. Our indebtedness to these brave men during the war is greater than we can ever hope to repay—to them we owe our safety from invasion and a great portion of our food supplies. Hospital Sunday this year seems to present a unique opportunity of showing our gratitude to the gallant men of the sea, who so nobly risked their lives in their country's cause. The greatest need at the present time is a sanatorium where patients suffering from tuberculosis may be cared for.

**South London Hospital for Women, South Side, Clapham Common, S.W. 4. Out-Patient Department, 86-90 Newington Causeway, S.E. 1.**—A general hospital for women and children staffed entirely by women doctors. During the year 1918 1,173 in-patients received treatment; of these, 273 were accommodated in the private wards of the hospital, which are proving a special boon to those whose incomes have been reduced as a result of the war, also to women workers, who in times of serious illness are unable to meet the expenses of operation, adequate medical attention, nursing, and the many necessities of a sick-room. £7,000 has to be obtained from voluntary contributions to meet the estimated expenditure for the current year. Contributions will be gratefully received by the Marchioness of Londonderry, chairman of the hospital.

**Victoria Hospital for Children, Chelsea, S.W.**—In addition to the ordinary upkeep of the institution, urgently required work in respect of renovating and cleaning, additional bedroom accommodation, and new heating system to replace present ancient and dilapidated one, must be executed without delay, at a cost of £11,000. Contributions should be sent to the Secretary, Mr. H. G. Evered.

**Westminster Hospital** was instituted in the year 1719, and thus has completed two hundred years of existence, during which it has administered to millions of the sick poor. During the war sick and wounded soldiers were received direct from overseas from the British Army (all branches), Canadian Forces, Australian Forces, South African Forces, and the Belgian Army. Many members of the medical and surgical staffs, and a large number of the nurses, have been engaged in war service at home and abroad. The great advance in wages and prices has hit the hospitals very severely, and a permanent addition of £10,000 to the pre-war income is urgently required.

